# Hyperprogressive disease in non-small cell lung cancer after PD-1/PD-L1 inhibitors immunotherapy: underlying killer

**DOI:** 10.3389/fimmu.2023.1200875

**Published:** 2023-05-22

**Authors:** Yanping Li, Tianhong Chen, Tian Yi Nie, Juyuan Han, Yunyan He, Xingxing Tang, Li Zhang

**Affiliations:** ^1^ Department of Respiratory Medicine, The Third People’s Hospital of Honghe Prefecture, Gejiu, China; ^2^ Department of Thoracic Surgery , The Third People’s Hospital of Honghe Prefecture, Gejiu, China; ^3^ Department of Thoracic Surgery, Yunnan Cancer Center, The Third Affiliated Hospital of Kunming Medical University, Kunming, China; ^4^ Department of Oncology, Gejiu City People’s Hospital, Diannan Central Hospital of Honghe Prefecture, The Fifth Affiliated Hospital of Kunming Medical University, Gejiu, China

**Keywords:** non-small cell lung cancer, PD-1/PD-L1, response pattern, hyperprogressive disease, immunotherapy

## Abstract

Immune checkpoint inhibitors (ICIs) target the negative regulatory pathway of T cells and effectively reactive the anti-tumor immune function of T cells by blocking the key pathway of the immune escape mechanism of the tumor—PD-1/PD-L1, and fundamentally changing the prospect of immunotherapy for non-small cell lung cancer patients. However, such promising immunotherapy is overshadowed by Hyperprogressive Disease, a response pattern associated with unwanted accelerated tumor growth and characterized by poor prognosis in a fraction of treated patients. This review comprehensively provides an overview of Hyperprogressive Disease in immune checkpoint inhibitor-based immunotherapy for non-small cell lung cancer including its definition, biomarkers, mechanisms, and treatment. A better understanding of the black side of immune checkpoint inhibitors therapy will provide a more profound insight into the pros and cons of immunotherapy.

## Introduction

**Figure 1:**
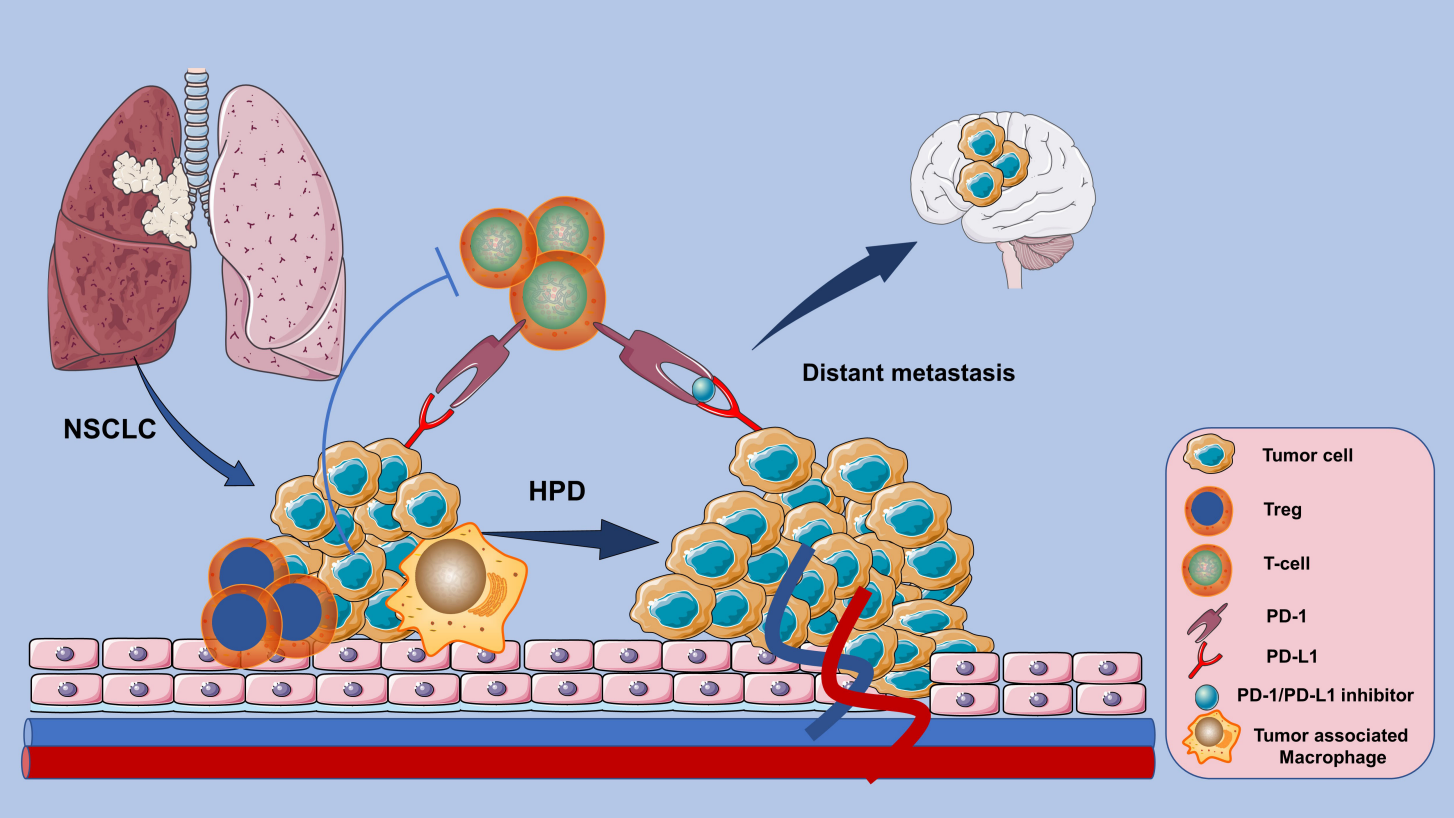
HPD response patterns associated with poor prognosis in NSCLC patients whose tumors instead accelerated in growth after PD-1/PD-L1 inhibitor-based immunotherapy.

Lung cancer is a serious life-threatening disease, and non-small cell lung cancer (NSCLC) is one of its most prevalent subtypes (1, 2). Immune checkpoint inhibitors (ICIs), as PD-1/PD-L1 inhibitors based immunotherapy has made revolutionized effects and become a milestone in the treatment history of NSCLC (3). However, PD-1/PD-L1 blockade can lead to an unsatisfactory response pattern characterized by accelerated tumor growth and associated with poor prognosis——Hyperprogressive Disease (HPD) (4). Detrimental patterns such as HPD and early death (ED) have been respectively observed in a proportion of NSCLC patients treated with ICIs (5). Overall survival (OS) is significantly reduced in NSCLC patients who develop HPD after PD-1/PD-L1 inhibitors blockade (4). For instance, although the PD-1 antibody Nivolumab is quite effective in clinical practice, HPD is not rare in patients with advanced NSCLC treated with Nivolumab and paralleled with a poor prognosis (6, 7). There are also several case reports about HPD events after treatment with another PD-1 inhibitor Pembrolizumab and the PD-L1 inhibitor - Durvalumab (8, 9). However, the definition and predictive biomarkers of HPD in NSCLC remain controversial, and the associated clinicopathological features or biological mechanisms are not yet determined. This significantly restricts the utilization of ICIs in patients with NSCLC (**Figure 1**).

## Definition of HPD in NSCLC immunotherapy

The accelerate growth in tumor size and volume measured by computed tomography(CT) during ICIs blockade are the most objective characteristics of HPD by using the Response Evaluation Criteria in Solid Tumors (RECIST) 1.1 criteria (10). However, the main limitation of conventional response assessment criteria RECIST 1.1 remains due to the inadequate ability to capture the response to immunotherapies and the inapplicability to patients without pre-baseline imaging or progression on unmeasurable lesions (11, 12). Therefore, novel criteria like iRECIST is clinically used as a response evaluation tool in patients undergoing immunotherapy (13). Despite the improvement, the definition of HPD has not been standardized and the prevalence of it varies based on different criteria (14) (**Table 1**). The assessment criteria should address the relevance of the clinical presentation, poor prognosis, and biological behavior of the NSCLC (21). The standard definition of HPD should be continuously optimized to guide better PD-1/PD-L1 inhibitors immunotherapy.

**Table 1:** The prevalence of HPD is varied based on different criteria in NSCLC.

Study	Incidence	Criteria	Conclusion	Ref
Ignacio Matos et al	12.5% (4/32)	HPD = 1.4 x baseline sum Target lesions Or HPD = 1.2 x baseline sum Target lesions + new lesions in at least two different organs	Capturing HPD by using RECIST criteria is intuitive and easy to implement.	(15)
	16.2% (1/27)	HPD = TGR experimental period/TGR reference period ≥ 2		
Youjin Kim et al	14.3% (48/135)	volumetry	volumetric measurement is more precise than the basis of one-dimensional analysis.	(16)
	13.1%(44/135)	RECIST 1.1		
Deirdre M.H.J. ten Berge et al	7%(4/58)	TGK	TGK has predictive value for OS	(17)
Roberto Ferrara et al	13.8%	ΔTGR exceeding 50%.	HPD is associated with high metastatic burden and poor prognosis	(4)
Baptiste Kas et al	18.5%(22/406)	the TGR ratio	ΔTGR>100 is close to the characteristics of HPD (increase of the tumor kinetics and poor survival).	(18)
	5.4%(75/406)	a progression pace >2-fold and TTF<2 months		
C G Kim et al	20.9%(55/263)	TGK	HPD meeting both TGK and TGR criteria is associated with worse PFS and OS	(19)
	20.5%(54/263)	TGR		
	37.3%(98/263)	TTF		
B Abbar et al	11.3%	TGRratio	TTF is the only indicator of significantly worsened OS.	(20)
	5.7%	ΔTGR		
	17.0%	TGK		
	9.6%	RECIST		
	31.7%	TTF		

PFS, progression-free survival; TGK, tumor growth kinetics; OS, overall survival; ΔTGR, The difference between TGR before and during therapy; TTF, time to treatment failure.

## Differentiating HPD from pseudoprogression

However, definitions based on radiological assessment alone have substantial technical limitations. The possibility of pseudoprogression (PsPD) exists when progressive deterioration of pulmonary infiltrative shadows is observed within 4 weeks among advanced NSCLC patients after the initial administration of anti-PD-1 antibody (22). Current clinical and radiological assessment strategies are inadequate to distinguish PsPD with HPD. PsPD has a similar response pattern of tumor increase or appearance of new lesions monitored by imaging at the beginning of treatment with ICIs, but shrinks later, whereas HPD is a rapid and poor prognosis progression pattern (23, 24). Consequently, repeat biopsies should be considered even if radiographic tumor progression is detected during immunotherapy (25–27). Besides, immune system-related response criteria such as NLR and ctDNA also have the potential to differentiate HPD from PsPD (28).

## Clinical characteristics and biomarkers for HPD

Conventional imaging methods are restricted to determine HPD. Image-based radiomic markers extracted from baseline CT of advanced NSCLC treated with PD-1/PD-L1 inhibitors including the features of peritumoral texture and nodule vessel-related tortuosity may have prospective value for identifying the HPD. Meanwhile, using radiomics features at the lesion-level analysis has the same effect. The novel radiomic models have translational implications to distinguish vulnerable NSCLC patients at risk of HPD (29–31).

In addition to imaging indices, sensitive predictive markers of positive and negative responses to immunotherapy and clinical factors that identify high-risk NSCLC populations that potentially progress to HPD after treatment with ICIs should be continuously developed (32, 33). There are lots of clinicopathological features associated with HPD in NSCLC patients treated with ICIs, as HPD was found associated with higher age (>65 years old) rather than higher tumor burden or specific tumor type (34) (**Table 2**). Subsequently, the related risk-predicting model based on clinical features is under exploring. Lung immune prognostic index (LIPI) based on dNLR > 3 and LDH > ULN is a promising tool for selecting patients who may not benefit from ICIs therapy (42).

**Table 2:** Clinicopathological features associated with HPD in NSCLC patients.

Study	Incidence	Risk factors	Ref
Yan Chen et al	8.02 to 30.43% (1389)	ECOG> 1, RMH score≥ 2, serum LDH level > ULN, the number of metastasis sites > 2, and liver metastasis	(35)
Yong Jun Choi et al	19.2% (15/78)	age, size of tumor and number of various metastatic lesions	(36)
Lee X Li et al	119/3129	elevated NLR	(37)
Youjin Kim et al	/	dNLR > 4 and LDH level > ULN	(16)
Jehun Kim et al	15.9%(35/219)	PD-L1 expression < 50%, metastatic sites≥ 3 NLR ≥ 3.3, and hemoglobin level < 10	(38)
Seo Ree Kim et al	11.3% (26/231)	heavy smoker, very low PD-L1 expression, multiple metastasis, and CAR index,	(39)
M P Petrova et al	4.8%(8/167)	a high pre-immunotherapy NLR2 and the presence of sarcopenia	(40)
Kristin L Ayers et al	/	African American patient group had lower incidence (14.7%) of HPD than the White patient group (24.5%).	(41)

ECOG, Eastern Cooperative Oncology Group; RMH score, Royal Marsden Hospital score; NLR, neutrophil-to-lymphocyte ratio; dNLR, derived neutrophil-to-lymphocyte ratio; ULN, upper limit of normal; LDH, lactate dehydrogenase; STK11, serine/threonine kinase 11 gene; ctDNA, circulating tumor DNA.

Genomic profiles is another key component of risk prediction models for HPD after immunotherapy. A case report illustrates that patients carrying EGFR exon 20 insertion and MYC amplification have the risk of developing to HPD after Nivolumab blockade (43). Besides, the coexistence of STK11 gene mutations and KRAS mutations can be used as potential biomarkers for HPD (16). Simultaneously, MDM2 family amplification or EGFR aberrations are closely linked with increasing TGR after PD-1/PD-L1 inhibitors monotherapy (44). Furthermore, long non-coding RNA (lncRNA) plays a critical role in the immune regulation of LUAD and the immune-related lncRNAs (IRLs) manifest a promising prediction value of ICIs efficacy in LUAD. Patients with low risk might gain benefits from ICIs whereas some have a risk of HPD (45). Additionally, Liquid biopsy could be assisted to identify patients at high risk of HPD, and ctDNA may be a novel prognostic biomarker of PD-1 blockade (46, 47).

There are more and more studies evaluating the predictive and prognostic value of the various immune cells in pretreatment tissue samples and identifying determinants associated with response in patients with NSCLC treated with ICIs. Levels of tumor-infiltrating lymphocytes (TILs) were strongly and independently associated with response to ICIs therapy (48). These studies illustrate that the different predictive and prognostic values for infiltrating immune cells in tumor tissue may help in selecting patients for ICIs. More importantly, the patient’s TILs assessment is relatively easy to incorporate into the pathology laboratory workflow, easy to perform and inexpensive. Besides, the analyzing of immune cell of PBMC gradually draws more and more attention (**Table 3**).

**Table 3:** Potential predictive immune biomarkers of HPD.

Study	Immune cell	Characteristics of TILs	Ref
C G Kim	CD8+ T lymphocytes	a lower frequency of effector/memory subsets (CCR7-CD45RA- T cells among the total CD8+ T cells) a higher frequency of severely exhausted populations (TIGIT+ T cells among PD-1+CD8+ T cells)	(19)
Kyung Hwan Kim		high pre-treatment frequency of CD39^+^CD8^+^ T cells	(49)
Hugo Arasanz	CD4+ T lymphocytes	A strong expansion of highly differentiated CD28^-^ CD4 T lymphocytes (CD4 THD) CD28^-^ CD4 T lymphocytes ≥ 1.3 (CD4 THD burst) was significantly associated with HPD	(50)
Giuseppe Lo Russo	Macrophages	infiltration by M2-like CD163^+^CD33^+^PD-L1^+^ clustered epithelioid macrophages.	(51)
Seo Ree Kim		fewer CD8+/PD-1+ TIL and more M2 macrophages in the tumor microenvironment	(39)

NLR, neutrophil-to-lymphocyte ratio; NLR1, neutrophil lymphocyte ratio; PLR1, platelet: lymphocyte ratio; PBMC, peripheral blood mononuclear cells; TAM, tumor-associated macrophages.

## Mechanism of HPD in NSCLC

Exploring the mechanisms of HPD in NSCLC is critical for understanding immunotherapy represented by ICIs. The tumour microenvironment (TME) is involved in influencing the response to immunotherapy as it plays a predominant role in the multiple interactions between tumor cells and the immune system (52). The biological basis and mechanisms of HPD are being elucidated and some studies have proposed immune checkpoint antibody-Fc/FcR interactions on macrophages as a mechanism of HPD after PD-1/PD-L1 blockade. Reprogramming of tumor associated macrophage (TAM) with the involvement of the Fc receptor of ICIs contribut to the induction of HPD (51). While, a study revealed that HPD was significantly linked with intratumoral B-cell density but not T-cell or macrophage (53). An animal model of a regulatory T cell (Treg)-dominated TME formed by selective depletion of CD8+ T cells by targeting CD8β antigen with near-infrared photoimmunotherapy (NIR-PIT) has shown that HPD after PD-1 blockade can be partly responsible for an imbalance between effector T cells and Tregs in the TME (54). Intrestingly, the interaction between the redox and immune system may lead to the local immunosuppression in the TME which accelerate tumor growth. Such as the administration of IgG4 and glutathione could promote tumor growth in the mouse lung cancer model (55).

Notably, analysis of the pathological features of patients who developed HPD during Pembrolizumab treatment for NSCLC suggests that the pathological type conversion of adenocarcinoma to small cell carcinoma may be the cause of HPD during ICIs treatment (56). Furthermore, changes in PD-L1 expression in tumor tissues may also be associated with HPD (8). It has been demonstrated that HPD can be prevented in preclinical models by targeting the IFNγ-PKM2-β-catenin axis. Tandem through the immunogenic, metabolic, and oncogenic pathway of the IFNγ-PKM2-β-catenin cascade is the primary mechanism of ICIs-associated HPD (57). There is an urgent need for further expansion of the scope of research and invasive research tools, and in-depth exploration of the underlying molecular mechanisms is of paramount importance.

## The management of HPD in NSCLC

A comprehensive and thorough study of the mechanisms involved not only provides a plausible explanation for HPD, but also offers new opportunities to manipulate this mechanism to improve cancer immunotherapy. PD-1 blockade may promote the proliferation of highly suppressive PD-1+ eTreg cells, leading to suppression of antitumor immunity and HPD. Therefore targeting depletion of eTreg cells in tumor tissue would be an effective strategy for the treatment and prevention of HPD (58). More importantly, salvage treatment after the onset of HPD in NSCLC is also under active investigation in clinical practice. Alternative therapies, like high-dose corticosteroids, antibiotics and drainage, can be effective in treating the symptoms of HPD caused by Nivolumab (59). Besides, termination of immunotherapy should be discussed after the onset of HPD is monitored and an early switch to cytotoxic therapy is essential to avoid further disease progression (60). For instance, a comparative study retrospectively screened patients with pathologically confirmed advanced or recurrent NSCLC demontrated that the HPD rate was significantly lower in the combination therapy (cytotoxic chemotherapy plus PD-1/PD-L1 inhibitor) group than in the PD-1/PD-L1 inhibitor monotherapy group (61). Chemotherapy has the value to increase a tumor’s response to immunotherapy and overcome the associated resistance (62). The combination therapy warrant further study to reduce the incidence of HPD. Moreover, informing patients of the risk of HPD is an indispensable component before the administration of ICIs. Health auhorities and trial sponsors are under obligation to monitor tumor progression in trials to help oncologists properly inform patients of the expected incidence of HPD.

## Discussion

Immunotherapy based on immune checkpoint inhibitors has brought revolutionary clinical benefits to patients with NSCLC, however, immunotherapy is also a double-edged sword that may bring about serious response patterns such as HPD, which deviates from the original intent of immunotherapy’s excellent clinical efficacy and high safety profile. The lack of consensus on the definitional criteria and biological basis of HPD necessitates larger studies and multicenter collaborations to standardize the criteria. How to maximize the efficacy and minimize the HPD caused by ICIS while consolidating existing therapeutic gains to benefit more NSCLC patients remains an open question. The importance of positive predictive markers for screening NSCLC patients who may benefit from immunotherapy with ICIs and the role of developing negative response predictive markers to screen out subgroups of NSCLC that do not benefit or may even develop HPD cannot be underestimated, therefor identifying potential molecular mechanisms and developing predictive biomarkers for HPD is an important direction.

## Author contributions

Conception and design: LZ, YL. Administrative support: TC, TN, JH. Provision of study materials: TC, XT. Collection and assembly of data: LZ, YH. Data analysis and interpretation: YL. Manuscript writing: All authors. Final approval of manuscript: All authors. All authors contributed to the article and approved the submitted version.
